# Supplementary material for: Nucleotide binding halts diffusion of the eukaryotic replicative helicase during activation
Source: Nat Commun. 2023 Apr 14;14:2082. doi: 10.1038/s41467-023-37093-9 (PMC10104875; doi:10.1038/s41467-023-37093-9)
Supplement: Supplementary file 1 — Supplementary Information [file 41467_2023_37093_MOESM1_ESM.pdf]

Supplementary Information for:

Nucleotide binding halts diffusion of the eukaryotic replicative helicase during activation

Daniel Ramírez Montero[1], Humberto Sánchez[1], Edo van Veen[1], Theo van Laar[1], Belén Solano[1], John F. X. Diffley[2]*, and Nynke H. Dekker[1]*.

[1]Department of Bionanoscience, Kavli Institute of Nanoscience, Delft University of Technology, Delft, The Netherlands.

[2] Chromosome Replication Laboratory, Francis Crick Institute, London, UK.

*Correspondence: john.diffley@crick.ac.uk; n.h.dekker@tudelft.nl

Supplementary Figures:

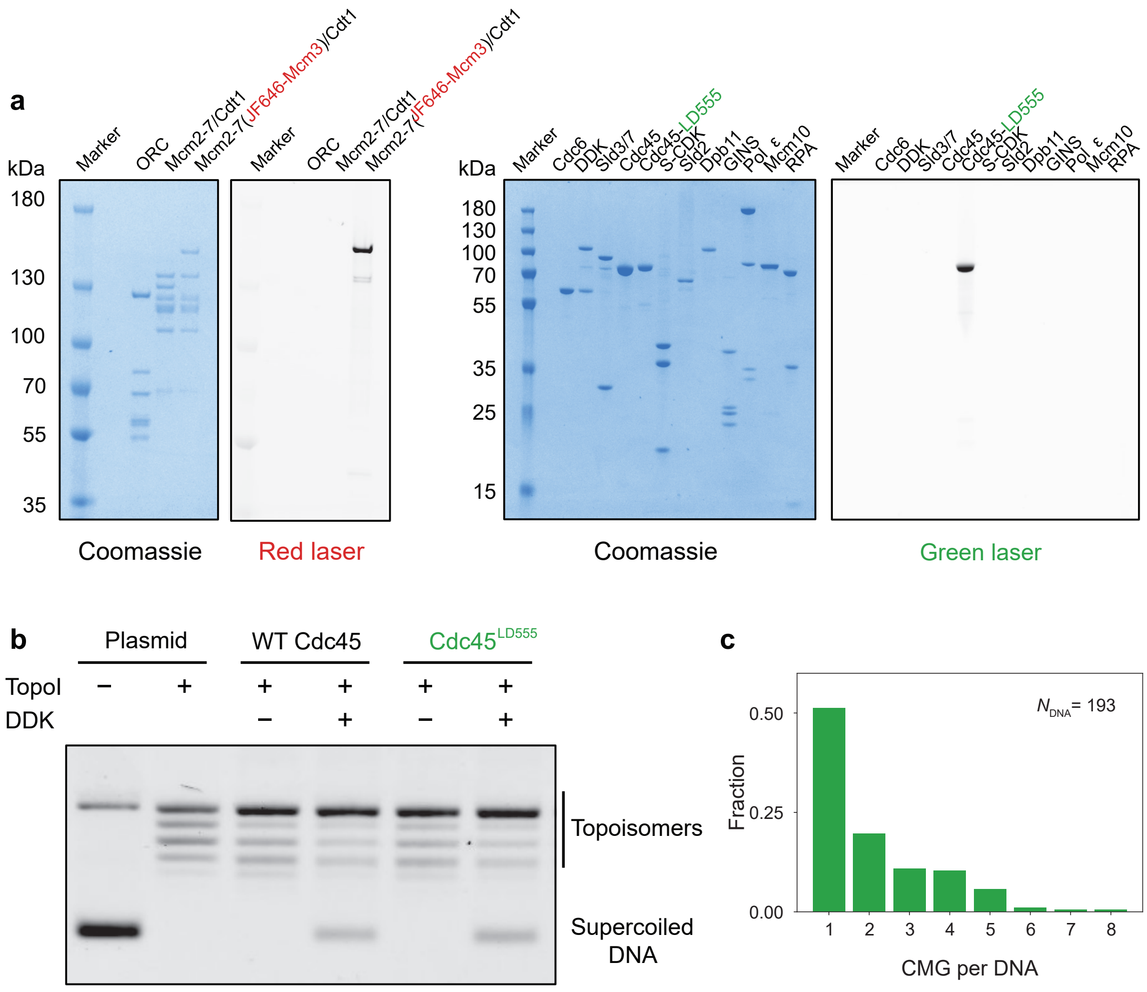

Supplementary Figure 1 | Hybrid ensemble and single-molecule assay and reagent validation. a, SDS-PAGE showing the minimal set of purified proteins required for the reconstitution of CMG assembly and activation; the gels were stained with Coomassie Blue Stain and fluorescently scanned with either a red or a green laser, to show the fluorescently labeled proteins in either color. **b**, Ensemble unwinding assay showing that Cdc45^{LD555} supports DNA unwinding to near WT levels (N=2 biological replicates). **c**, Distribution of total numbers of fluorescent CMG complexes per DNA, obtained by combining the total number of CMG diffraction-limited spots per DNA (Fig. 1b) with the number of CMG complexes within each spot (Fig. 1c). Source data are provided as a Source Data file.

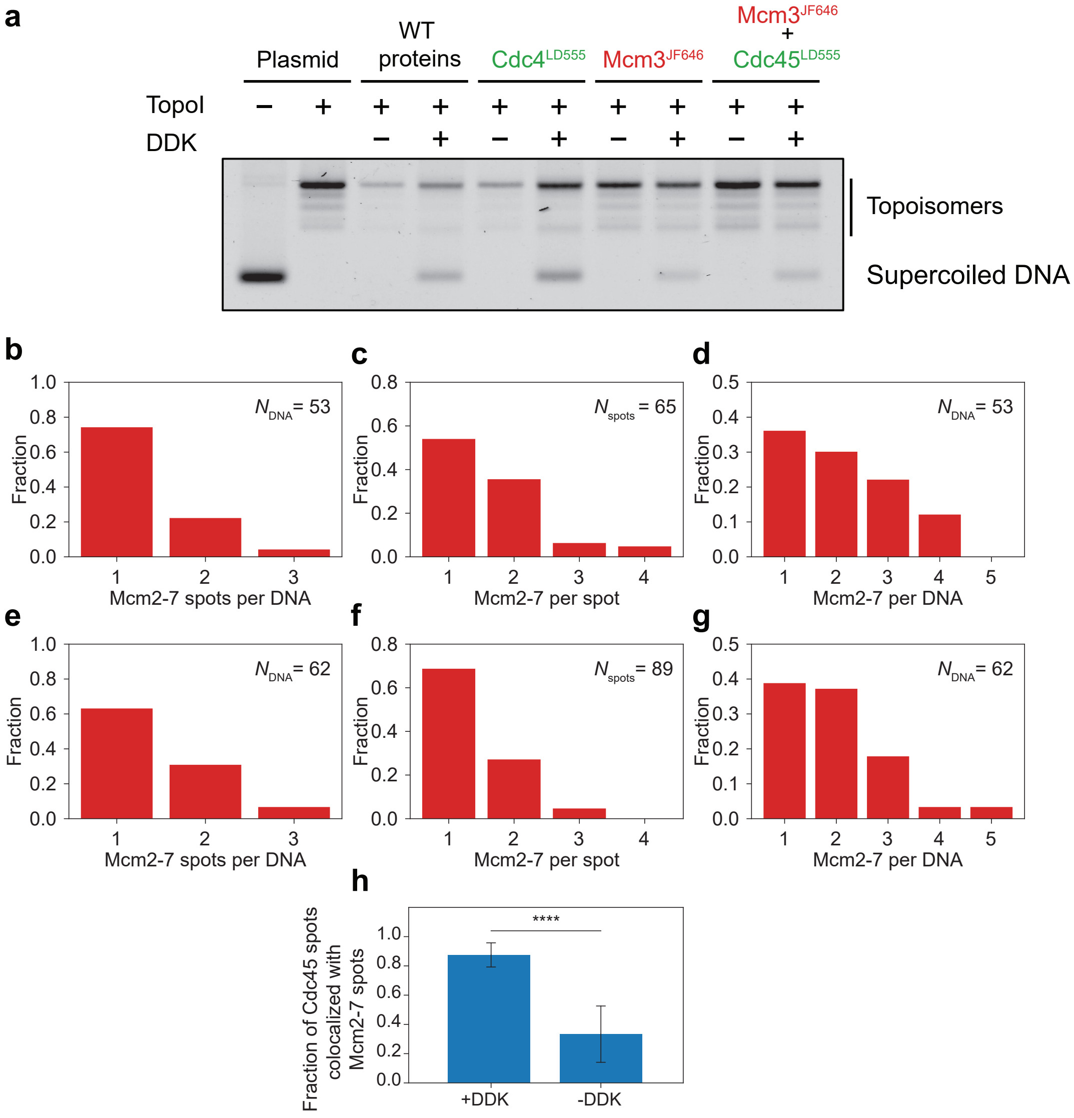

Supplementary Figure 2 | Reagent validation, distribution of number of Mcm2-7 spots and distribution of Mcm2-7 complexes within each spot. a, Ensemble unwinding assay showing that Mcm2-7^{JF646} supports DNA unwinding alone and in conjunction with Cdc45^{LD555} (N=1 biological replicate). **b**, Distribution of the number of Mcm2-7 diffraction-limited spots per DNA in the presence of DDK. **c**, Distribution of the number of Mcm2-7 complexes within each diffraction-limited spot in the presence of DDK. **d**, Distribution of the total number of Mcm2-7 complexes per DNA molecule in the presence of DDK, obtained by combining data from **b** and **c**. **e**, Distribution of the number of Mcm2-7 diffraction-limited spots per DNA in the absence of DDK. **f**, Distribution of the number of Mcm2-7 complexes within each diffraction-limited spot in the absence of DDK. **g**, Distribution of the total number of Mcm2-7 complexes per DNA molecule in the absence of DDK, obtained by combining data from **e** and **f**. **h**, Mean fraction of Cdc45^{LD555} diffraction-limited spots that are colocalized with Mcm2-7^{JF646} diffraction-limited spots in the presence ($N_{Cdc45\ spots}$=16) or absence ($N_{Cdc45\ spots}$=6) of DDK; error bars show the standard error of proportion. Statistical significance was obtained from a two-sided binomial test (p-value=1.2×10^{-5}). Source data are provided as a Source Data file.

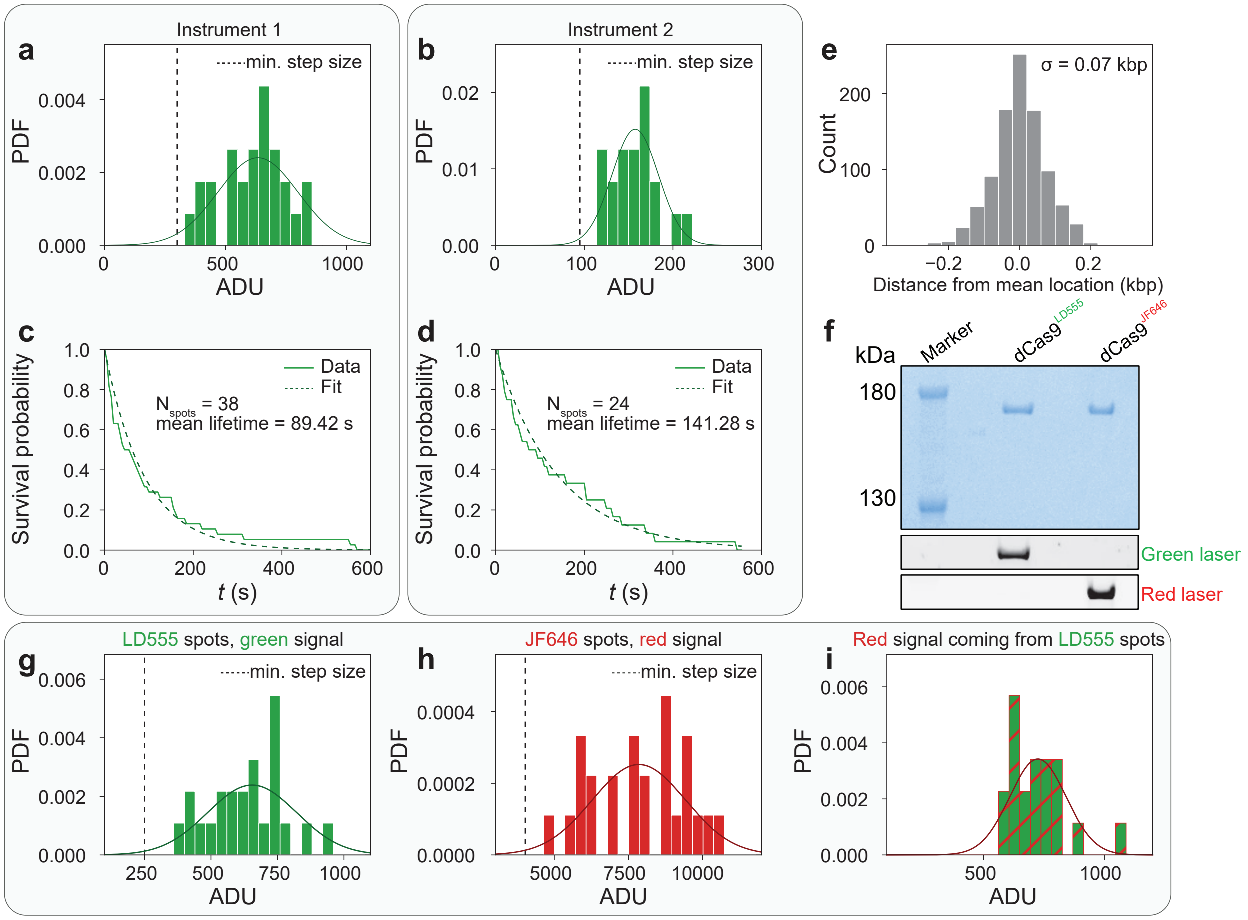

j

Instrument	fluorophore	signal	ΔI_{min} (ADU)	$\mu_{\Delta I}$ (ADU)	$\sigma_{\Delta I}$ (ADU)	N_{spots}
1	LD555	g	300	634	166	27
2	LD555	g	95	157	26.3	20
1	LD555	g	250	655	167	24
1	LD555	r (crosstalk)	-	725	117	20
1	JF646	r	4.00×10^3	7.85×10^3	1.58×10^3	25
1	JF646	g (crosstalk)	-	negligible	negligible	-

Supplementary Figure 3 | Fluorescently labeled dCas9 proteins as standards for determination of number of proteins per diffraction-limited spot and localization accuracy. a-b, Distribution of photobleaching step sizes of fluorescently labeled dCas9^{LD555} imaged under the same imaging conditions as fluorescent CMG in the single-color experiments; **a**, and **b**, correspond to the two instruments used in this study; both distributions were fitted to a normal distribution; $\mu - 2\sigma$ was used as the minimum step size in the single-color CMG experiments to capture at least 95 % of bleaching events. **c-d**, Distribution of times to photobleaching of fluorescently labeled dCas9^{LD555} imaged under the same imaging conditions as fluorescent CMG; **c**, and **d**, correspond to the two instruments used in this study; both distributions were fitted to a single exponential decay. **e**, distribution of positional measurements of fluorescently labeled dCas9^{LD555}; as dCas9^{LD555} is expected to be static, the standard deviation of this distribution gives us the localization error in our experiments. **f**, SDS-PAGE of dCas9 with fluorescently labeled with dyes LD555, JF646, respectively; the gel was stained with Coomasie Blue stain and fluorescently scanned with a red, green laser, respectively. **g**, Distribution of photobleaching step sizes of fluorescently labeled dCas9^{LD555} when simultaneously excited with the green and red lasers in instrument 1, as done in the Mcm2-7 and Cdc45 colocalization experiments; the distribution was fitted to a normal distribution; $\mu - 2\sigma$ was used as the minimum step size in the dual-color CMG experiments to capture at least 95 % of bleaching events. **h**, Distribution of photobleaching step sizes of fluorescently labeled dCas9^{JF646} when simultaneously excited with the green and red lasers in instrument 1, as done in the Mcm2-7 and Cdc45 colocalization experiments; the distribution was fitted to a normal distribution; $\mu - 2\sigma$ was used as the minimum step size in the dual-color CMG experiments to capture at least 95 % of bleaching events. **i**, Distribution of red signal coming from green fluorescently labeled dCas9^{LD555} when simultaneously excited with the green and red lasers in instrument 1, as done in the Mcm2-7 and Cdc45 colocalization experiments. The distribution was fitted to a normal distribution and the mean value was used for crosstalk corrections. **j**, Summary table of all the parameters obtained from **a-e**, and **g-i**. Source data are provided as a Source Data file.

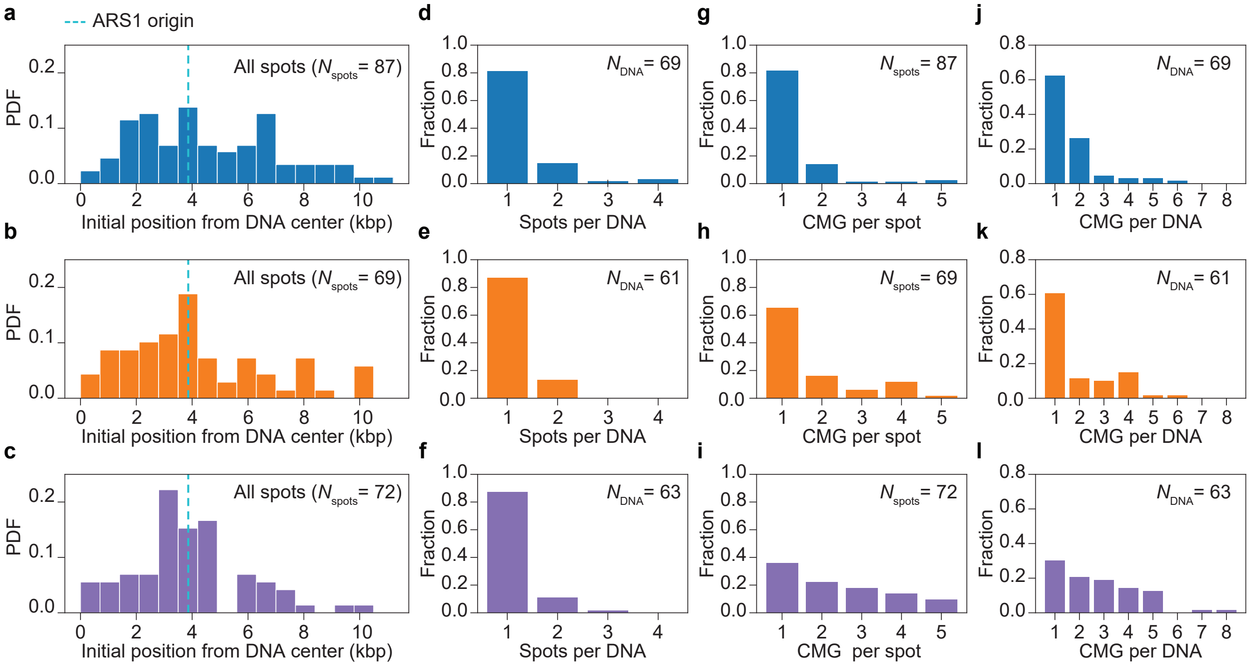

Supplementary Figure 4 | Distribution of initial positions, numbers of CMG spots and numbers of CMG complexes within each spot for the different biochemical conditions tested. a-c, Distribution of initial positions on the DNA of all Cdc45 diffraction-limited spots for DNA molecules imaged in **a**, the presence of ATP, **b**, the absence of nucleotide or, **c**, the presence of ATPγS. **d-f**, Distribution of numbers of CMG diffraction-limited spots for DNA molecules imaged in **d**, the presence of ATP, **e**, the absence of nucleotide or, **f**, the presence of ATPγS. **g-i**, Distribution of numbers of CMG complexes within each diffraction limited spot on DNA molecules imaged in **g**, the presence of ATP, **h**, the absence of nucleotide or, **i**, the presence of ATPγS. **j-l**, Distribution of numbers of CMG complexes per DNA for DNA molecules imaged in **j**, the presence of ATP, **k**, the absence of nucleotide or, **l**, the presence of ATPγS.

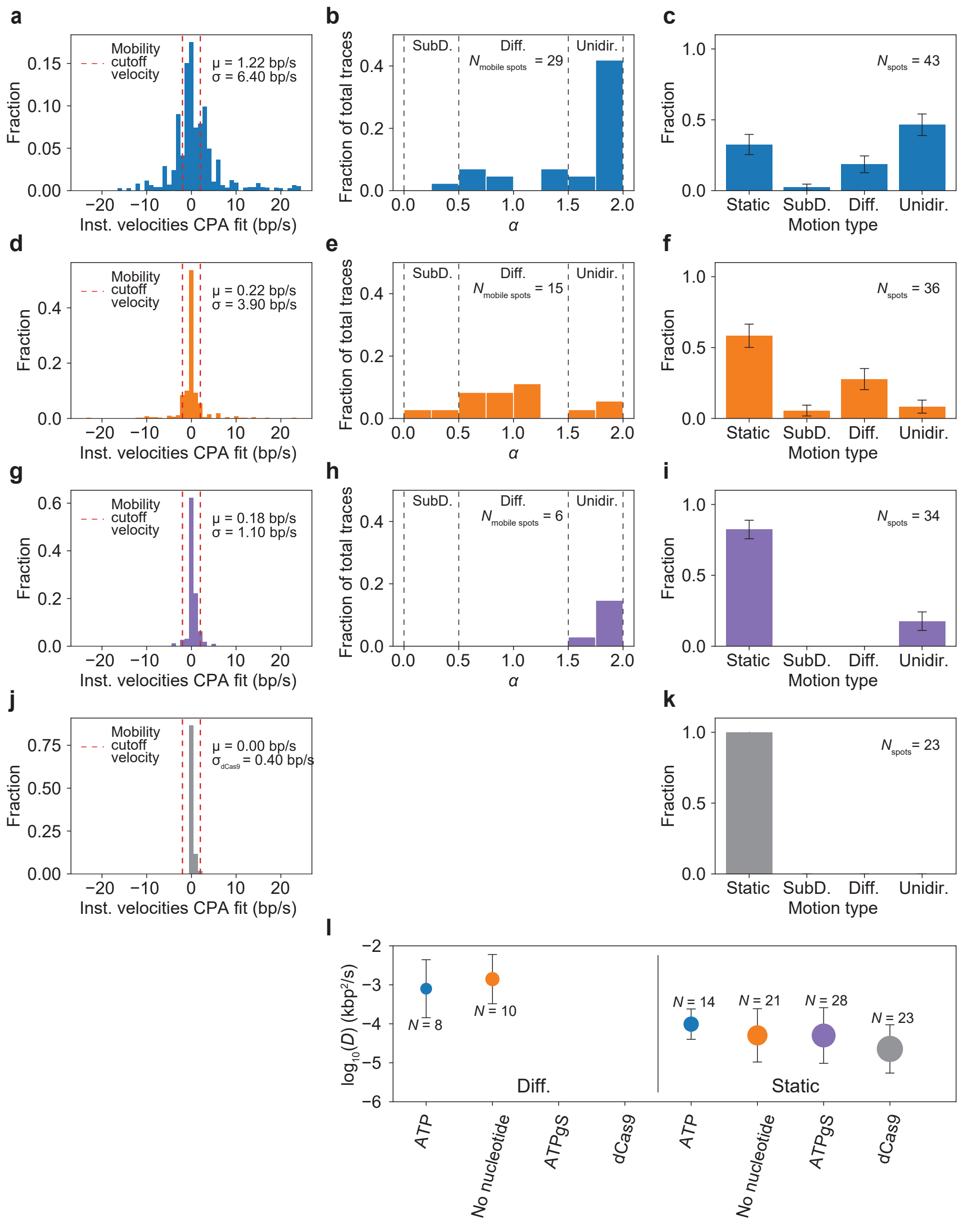

Supplementary Figure 5 | Mobility determination and motion classification of fluorescent spots imaged under different biochemical conditions. a, Distribution of instantaneous velocities coming from the CPA fits of CMG spots in the presence of ATP; red lines show the instantaneous velocity cutoff ($5\sigma_{dCas9}$) used to separate CMG spots into static or mobile. **b,** Distribution of anomalous coefficients α of mobile CMG spots in the presence of ATP. **c,** Fraction of CMG spots imaged in the presence of ATP classified into static, subdiffusive, diffusive or unidirectionally moving (N_{spots}=43); error bars show the standard error of proportion. **d,** Distribution of instantaneous velocities coming from the CPA fits of CMG spots in the absence of nucleotide; red lines show the instantaneous velocity cutoff ($5\sigma_{dCas9}$) used to separate CMG spots into static or mobile. **e,** Distribution of anomalous coefficients α of mobile CMG spots in the absence of nucleotide. **f,** Fraction of CMG spots imaged in the absence of of nucleotide classified into static, subdiffusive, diffusive or unidirectionally moving (N_{spots}=36); error bars show the standard error of proportion. **g,** Distribution of instantaneous velocities coming from the CPA fits of CMG spots in the presence of ATPγS; red lines show the instantaneous velocity cutoff ($5\sigma_{dCas9}$) used to separate CMG spots into static or mobile. **h,** Distribution of anomalous coefficients α of mobile CMG spots in the presence of ATPγS. **i,** Fraction of CMG spots imaged in the presence of ATPγS classified into static, subdiffusive, diffusive or unidirectionally moving (N_{spots}=34); error bars show the standard error of proportion. **j,** (*same as inset in Fig. 2a*) Distribution of instantaneous velocities coming from the CPA fits of dCas9^{LD555} spots; red lines show the instantaneous velocity cutoff ($5\sigma_{dCas9}$) used to separate CMG spots into static or mobile. **k,** Fraction of dCas9^{LD555} spots classified into static, subdiffusive, diffusive or unidirectionally moving (N_{spots}=23). **l,** (left half) Diffusion constants of spots classified as diffusive for the different biochemical conditions tested (mean *D* +/− standard deviation); (right half) Diffusion constants of spots classified as static for the different biochemical conditions tested (mean *D* +/− standard deviation).

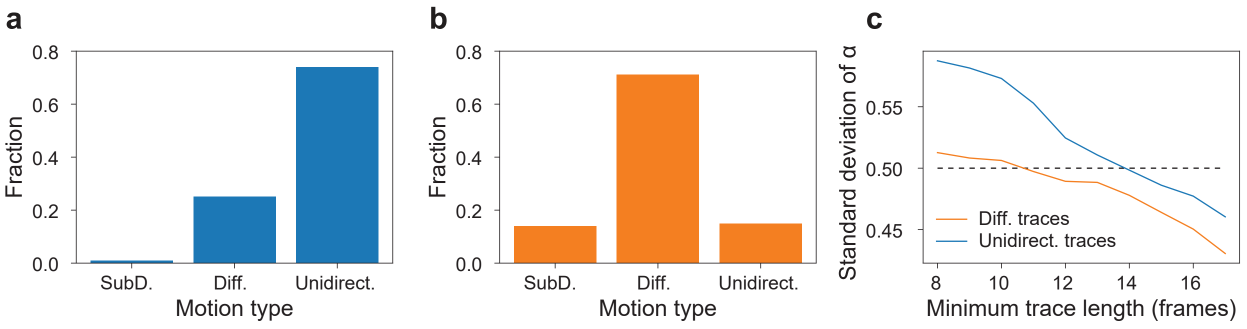

Supplementary Figure 6 | Motion classification of simulated unidirectional or diffusive traces and anomalous diffusion exponent error determination. Motion classification of simulated **a**, unidirectionally translocating traces with a representative velocity (5 bp/s) and **b**, diffusive traces with a representative diffusion coefficient (1.5×10^{-3} kb^2/s). **c**, Error determination of the anomalous diffusion exponent α as a function of the minimum trace length; the error falls below 0.5 for a minimum trace length of 14 frames. We start with 512 traces of each motion type with a minimum trace length of 8 pulled from a population with a mean fluorophore lifetime of 25 frames, and gradually increase the trace length filtering. The traces used in **a-b**, are those with a minimum trace length of 14, to mirror the motion analysis done on experimentally obtained CMG spots.

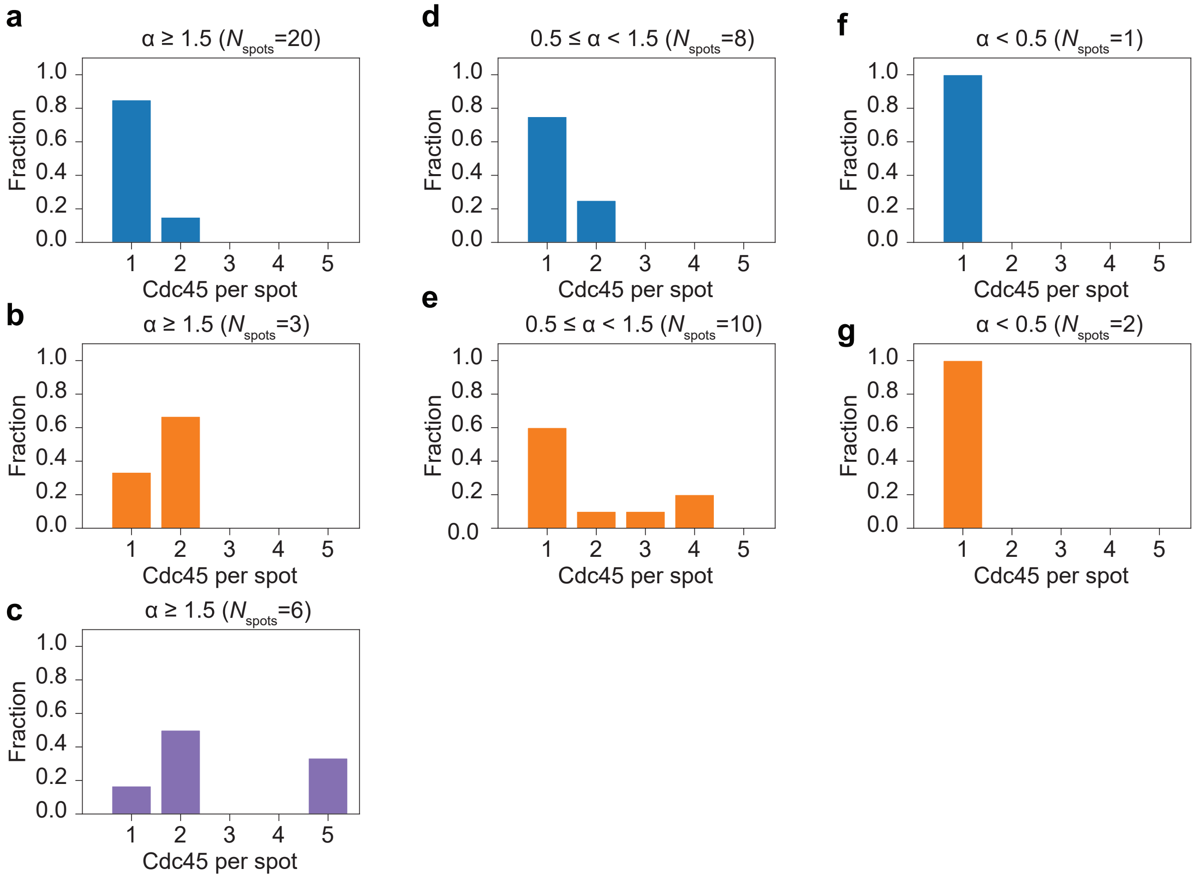

Supplementary Figure 7 | Distribution of number of Cdc45 molecules per mobile diffraction-limited spot for the different biochemical conditions tested. a-c, Distribution of number of Cdc45 molecules within diffraction-limited spots classified as unidirectionally-moving in the **a,** presence of ATP, **b,** absence of nucleotide or **c,** presence of ATPγS. **d-e,** Distribution of number of Cdc45 molecules within diffraction-limited spots classified as diffusive in the **d,** presence of ATP or **e,** absence of nucleotide. **f-g,** Distribution of number of Cdc45 molecules within diffraction-limited spots classified as subdiffusive in the **f,** presence of ATP or **g,** absence of nucleotide.

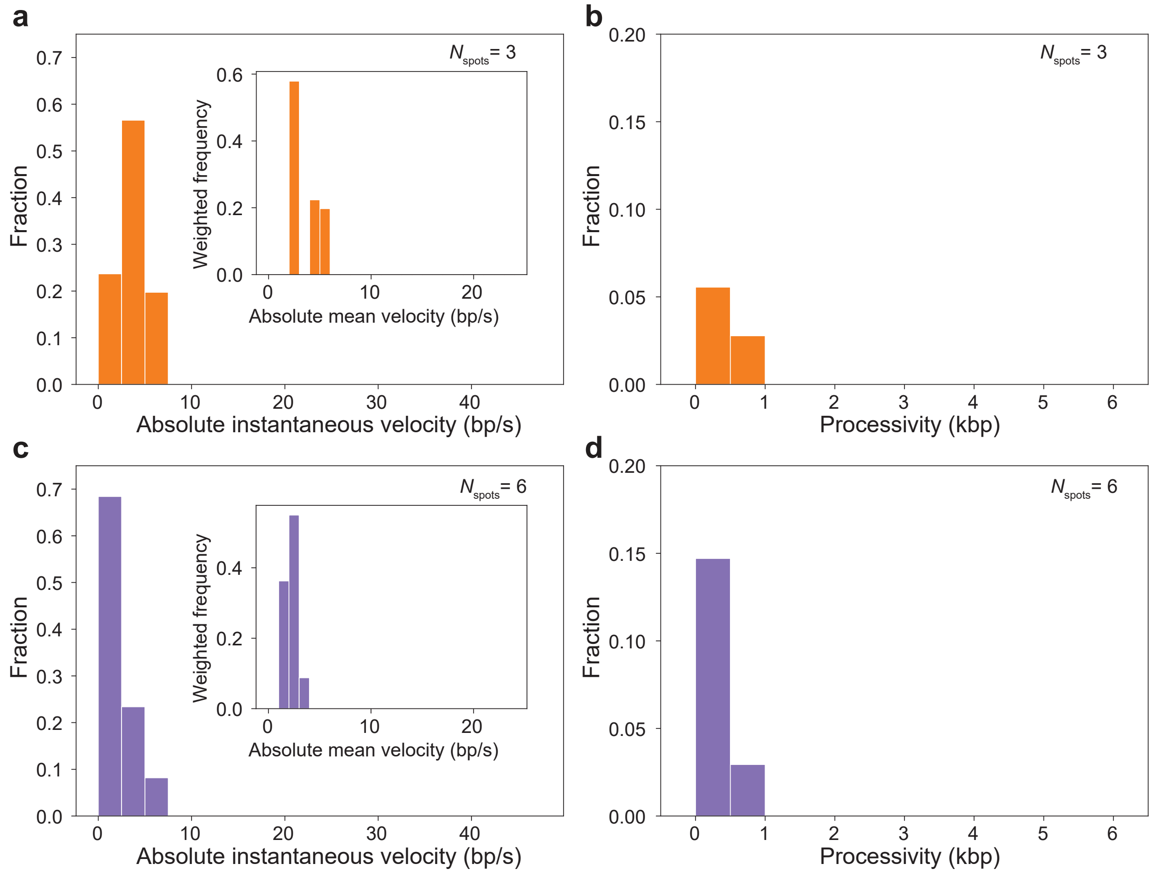

Supplementary Figure 8 | Analysis of unidirectionally moving CMG under different biochemical conditions. a, Distribution of absolute instantaneous velocities of unidirectionally moving CMG spots in the absence of nucleotide; (inset) Distribution of absolute mean velocities of unidirectionally moving CMG spots in the absence of nucleotide normalized by the length of each trace. **b,** Distribution of processivities of unidirectionally moving CMG spots in the absence of nucleotide. **c,** Distribution of absolute instantaneous velocities of unidirectionally moving CMG spots in the presence of ATPγS; (inset) Distribution of absolute mean velocities of unidirectionally moving CMG spots in the presence of ATPγS normalized by the length of each trace. **d,** Distribution of processivities of unidirectionally moving CMG spots in the presence of ATPγS.

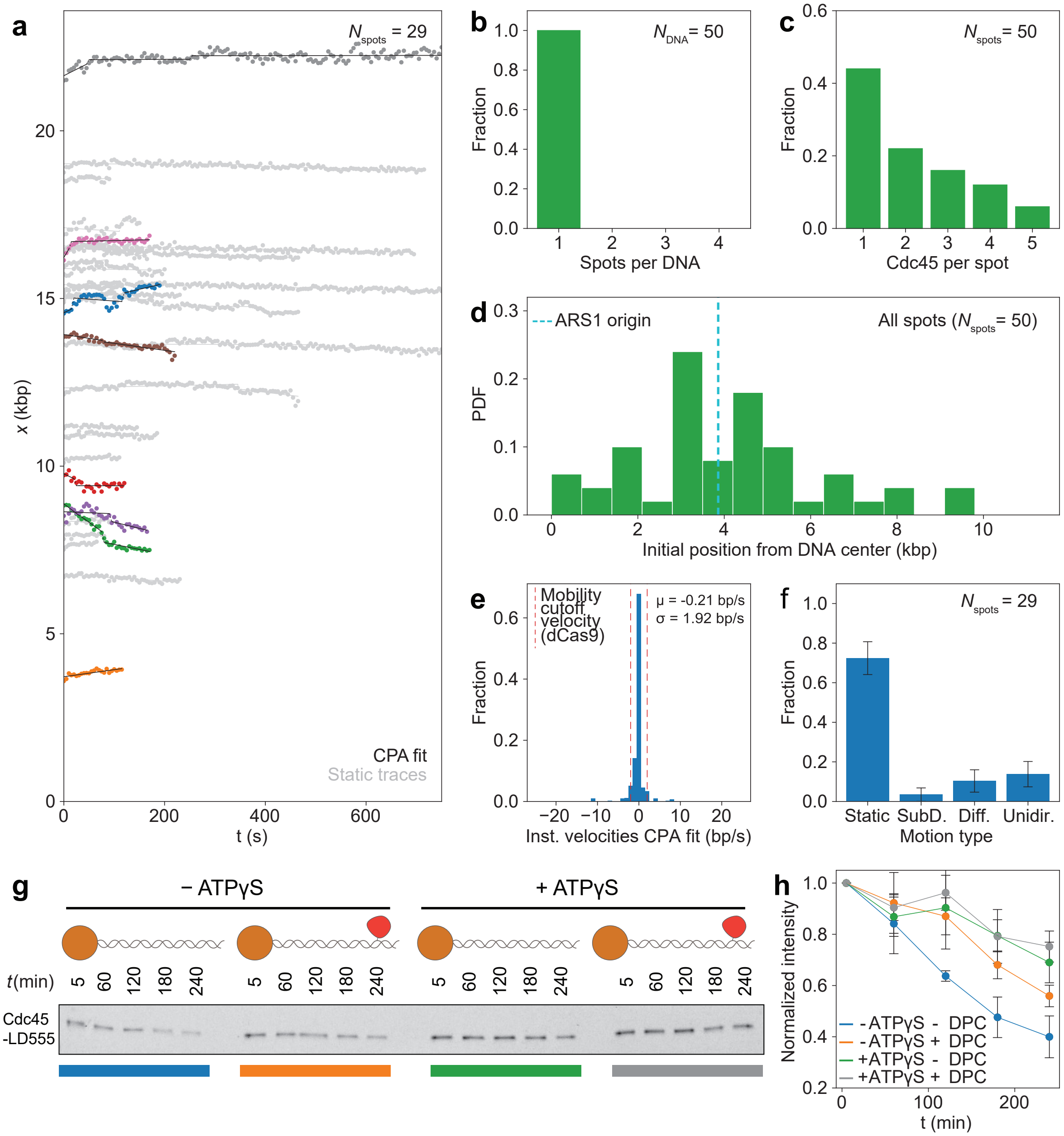

Supplementary Figure 9 | Nucleotide binding halts CMG diffusion independently of DNA melting. **a**, Position *vs.* time plots of CMG$^{Mcm2(6A)}$ spots in the presence of ATP; CPA fits are plotted in black, static traces are shown in light gray and mobile traces are shown in all other colors. **b**, Distribution of numbers of CMG$^{Mcm2(6A)}$ diffraction-limited spots per DNA. **c**, Distribution of numbers of CMG$^{Mcm2(6A)}$ complexes within each diffraction-limited spot. **d**, Distribution of initial positions on the DNA of all CMG$^{Mcm2(6A)}$ diffraction-limited spots. **e,** Distribution of instantaneous velocities coming from the CPA fits of CMG$^{Mcm2(6A)}$ spots in the presence of ATP; red lines show the instantaneous velocity cutoff ($5\sigma_{dCas9}$) used to separate CMG$^{Mcm2(6A)}$ spots into static or mobile. **f,** Fraction of CMG$^{Mcm2(6A)}$ spots imaged in the presence of ATP classified into static, subdiffusive, diffusive or unidirectionally moving (N_{spots}=29); error bars show the standard error of proportion. **g**, Fluorescent scan of an SDS-PAGE gel showing the amount of Cdc45^{LD555} left on linear DNA bound to magnetic beads at one end and containing either a free end or an end capped with a covalently crosslinked methyltransferase. **h**, Densitometry quantification of the experiment shown in **g**, showing the average normalized intensity of three replicates together with their standard deviation. Data points are connected by solid lines to guide the eye. Source Data are provided as a Source Data file.

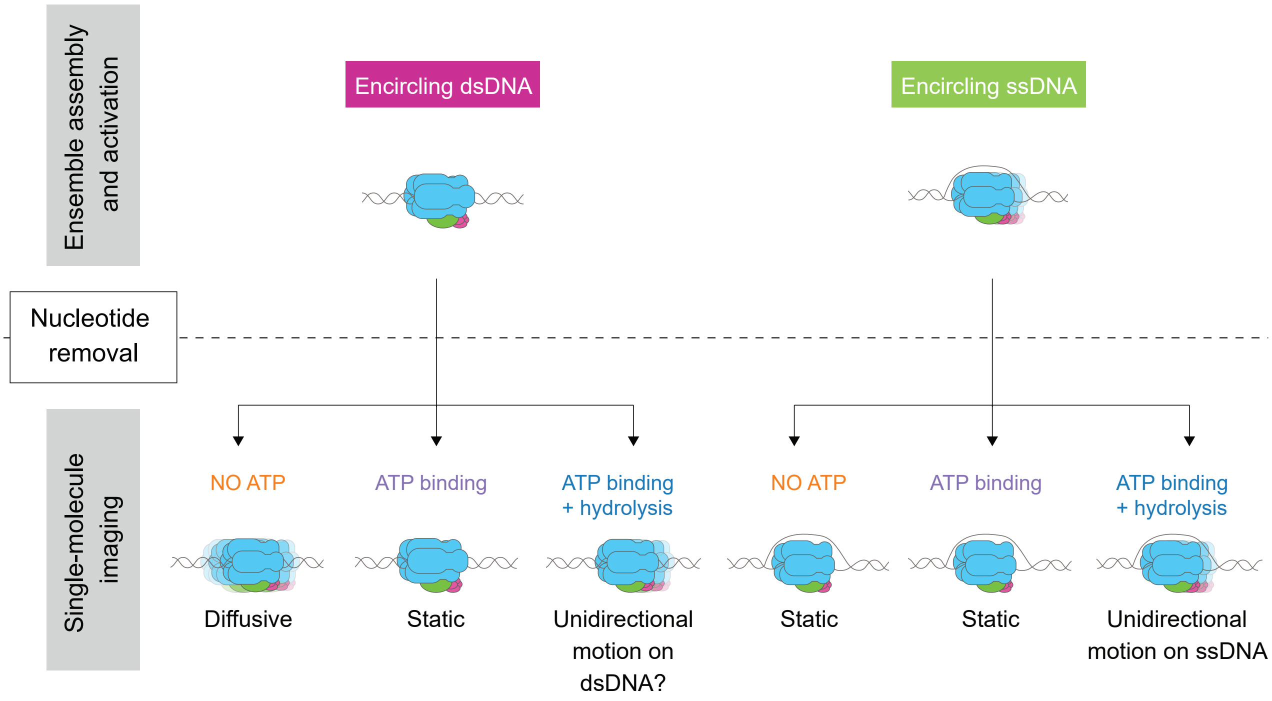

Supplementary Figure 10 | Final model. Model showing all the experimental outcomes observed in this study with different potential explanations.

Supplementary Tables:

Supplementary Table 1: oligos and primers used in this study

Name	5' to 3' sequence
DRM_005	GCTGCGCCTGCTGAACGGTGATTATAAAGATGATGATGGG
DRM_006	AGCCAGCTCAGGCTATCGCCCTCGTCTGTGACTTCATC
DRM_184	ACGGCTGTTAAATGGGGGGAGTGATAAGAAATACTCAATAGGC
DRM_185	AATAACCAACTTAATGAATCCCCCACGTGATGATGATGATG
TL_033	GCGCGCCAATTGGAGCTCCACCGCGG
TL_034	GGCGCGCCGGAAACAGCTATGACCATGATTACGCC
DRM_218	ATACTTTAGATTGATTTC[5-Fluoro-2'-dC]GGCTTCACCTG
DRM_220	ATACTTTAGATTGATTTCCGGCTTCACCTG
DRM_222	Biotin-CTAGTGGATCCCCAGGGCT

Supplementary Table 2: gBlocks™ used in this study

**gBlock™**	**5' to 3' Sequence**
gBlock™ DRM8	TCATTCTGAGAATAGTGTATGCGGCGACCGAGTTGCTCTTGCCTGGCGTCAATAC<br>GGGATAATACCGCGCCACATAGCAGAACTTTAAAAGTGCTCATCATTGGAAAACGT<br>TCTTCGGGGCGAAAACTCTCAAGGATCTTACCGCTGTTGAGATCCAGTTCGATGTA<br>ACCCACTCGTGCACCCAACTGATCTTCAGCATCTTTTACTTTCACCAGCGTTTCTG<br>GGTGAGCAAAAACAGGAAGGCAAAATGCCGCAAAAAAGGGAATAAGGGCGACAC<br>GGAAATGTTGAATACTCATACTCTTCCTTTTTCAATATTATTGAAGCATTTATCAGG<br>GTTATTGTCTCATGAGCGGATACATATTTGAATGTATTTAGAAAAATAAACAAATAG<br>GGGTTCCGCGCACATTTCCCCGAAAAGTGCCACCTAAATTGTAAGCGTTAATATTT<br>TGTTAAAATTCGCGTTAAATTTTTGTTAAATCAGCTCATTTTTTAACCAATAGGCCGA<br>AATCGGCATAATCCCTTATAAATCAAAAGAATAGACCGAGATAGGGTTGAGTGTTG<br>TTCCAGTTTGGAACAAGAGTCCACTATTAAAGAACGTGGACTCCAACGTCAAAGGG<br>CGAAAAACCGTCTATCAGGGCGATGGCCCACTACGTGAACCATCACCCTAATCAA<br>GTTTTTTGGGGTCGAGGTGCCGTAAAGCACTAAATCGGAACCCTAAAGGGAGCCC<br>CCGATTTAGAGCTTGACGGGGAAAGCCCGCGAACGTGGCGAGAAAGGAAGGGAA<br>GAAAGCGAAAGGAGCGGGCGCTAGGGCGCTGGCAAGTGTAGCGGTCACGCTGC<br>GCGTAACCACCACACCCGCCGCGCTTAATGCGCCGCTACAGGGCGCGTCCCATT<br>CGCCATTGCTGAGGCGCAACTGTTGGGAAGGGCGATCGGTGCGGGCCTCTTCGC<br>TATTACGCCAGCTGGCGAAAGGGGGATGTGCTGCAAGGCGATTAAGTTGGGTAAC<br>GCCAGGGTTTTCCCAGTCACGACGTTGTAAAACGACGGCCAGTGAATTGTAATAC<br>GACTCACTATAGGGCGAATTGGAGCTCCACCGCGGTGGCGGCCGCTCTAGAACT<br>AGTGGATCCCCAGGGCTGCAGGAATTCGAGCTCGGTACCCACAATCAATCAAAAA<br>GCCAAATGATTTAGCATTATCTTTACATCTTGTTATTTTACAGATTTTATGTTTAGAT<br>CTTTTATGCTTGCTTTTCAAAAGGCCTGCAGGCAAGTGCACAAACAATACTTAAATA<br>AATACTACTCAGTAATAACCTATTTCTTAGCATTTTTGACGAAATTTGCTATTTTGTT<br>AGAGTGGGGATCCTCTAGAGTCGACCTGCAGGCATGCAAGCTTATCGATACCGTC<br>GACCTCGAGGGGGGGCACGGTACCAGCTTTTGTTCCCTTTAGTGAGGGTTAATTT<br>CGAGCTTGGCGTAATCATGGTCATAGCTGTTTCCTGTGTGAAATTGTTATCCGCTC<br>ACAATTCCACACAACATACGAGCCTGAAGCATAAAGTGTAAAGCCTGGGGTGCCT<br>AATGAGTGAGCTAACTCACAACCTCAGCTTGCGCTCACTGCCCGCTTTCCAGTCG<br>GGAAACCTGTCGTGCCAGCTGCATTAATGAATCGGCCAACGCGCGGGGAGAGGC<br>GGTTTGCGTATTGGGCGCTCTTCCGCTTCCTCGCTCACTGACTCGCTGCGCTCGG<br>TCGTTCGGCTGCGGCGAGCGGTATCAGCTCACTCAAAGGCGGTAATACGGTTATC<br>CACAGAATCAGGGGATAACGCAGGAAAGAACATGTGAGCAAAAGGCCAGCAAAAG<br>GCCAGGAACCGTAAAAAGGCCGCGTTGCTGGCGTTTTTCCATAGGCTCCGCCCCC<br>CTGACGAGCATCACAAAAATCGACGCTCAAGTCAGAGGTGGCGAAACCCGACAGG<br>ACTATAAAGATACCAGGCGTTTCCCCCTGGAAGCTCCCTCGTGCGCTCTCCTGTTC<br>CGACCCTGCCGCTTAACGGATACCTGTCCGCCTTTCTCCCTTCGGGAAGCGTGGC<br>GCTTTCTCATAGCTCACGCTGTAGGTATCTCAGTTCGGTGTAGGTCGTTCGCTCCA<br>AGCTGGGCTGTGTGCACGAAAACAGGATTAGCAGAGCGAGGTATGTAGGCGGTG<br>CTACAGAGTTCTTGAAGTGGTGGCCTAACTACGGCTACACTAGAAGGACAGTATTT<br>GGTATCTGCGCTCTGCTGAAGCCAGTTACCTTCGGAAAAAGAGTTGGTAGCTCTT<br>GATTCGGCAAACATACCAACGCTGGTAGCGGTAGTATTTTTGTTTGCAAGCAGCAG<br>ATTACGCGCAGAAAAAAAGGATCTCAAGATGATCCTTTGATCTTTTCTACGGGGTC<br>TGACGCTCAGTGGAACGAAAACTCACGTTAAGGGATATTGGTCATGAGATTATCAA<br>AATGGATCTTCACCTAGATCCTTTTAAATTACAGGTGAAGCCGGAAATCAATCTAAA<br>GTAT
