# Supplementary material for: Nucleotide binding halts diffusion of the eukaryotic replicative helicase during activation
Source: Nat Commun. 2023 Apr 14;14:2082. doi: 10.1038/s41467-023-37093-9 (PMC10104875; doi:10.1038/s41467-023-37093-9)
Supplement: Supplementary file 3 — Description of Additional Supplementary Files [file 41467_2023_37093_MOESM3_ESM.pdf]

Description of Additional Supplementary Files

File Name: Supplementary Movie 1
Description: Example of diffraction-limited spot of fluorescent CMG moving unidirectionally along a DNA molecule held in an optical trap in the presence of ATP.

File Name: Supplementary Movie 2
Description: Example of a diffraction-limited spot of fluorescent CMG diffusing along a DNA molecule held in an optical trap in the absence of ATP.

File Name: Supplementary Movie 3
Description: Example of two unidirectionally moving fluorescent CMGs that start within the same diffraction-limited spot and split up into two distinct diffraction-limited spots that move in opposite directions along a DNA molecule held in an optical trap in the presence of ATP (related to Fig. 3c).
