# Supplementary material for: Nucleotide binding halts diffusion of the eukaryotic replicative helicase during activation
Source: Nat Commun. 2023 Apr 14;14:2082. doi: 10.1038/s41467-023-37093-9 (PMC10104875; doi:10.1038/s41467-023-37093-9)
Supplement: Supplementary file 7 — Reporting Summary [file 41467_2023_37093_MOESM7_ESM.pdf]

nature portfolio

Corresponding author(s): John F. X. Diffley, Nynke H. Dekker

Last updated by author(s): Feb 1, 2023

Reporting Summary

Nature Portfolio wishes to improve the reproducibility of the work that we publish. This form provides structure for consistency and transparency in reporting. For further information on Nature Portfolio policies, see our Editorial Policies and the Editorial Policy Checklist.

Statistics

For all statistical analyses, confirm that the following items are present in the figure legend, table legend, main text, or Methods section.

n/a	Confirmed	
☐	☒	The exact sample size (*n*) for each experimental group/condition, given as a discrete number and unit of measurement
☐	☒	A statement on whether measurements were taken from distinct samples or whether the same sample was measured repeatedly
☐	☒	The statistical test(s) used AND whether they are one- or two-sided<br>*Only common tests should be described solely by name; describe more complex techniques in the Methods section.*
☒	☐	A description of all covariates tested
☒	☐	A description of any assumptions or corrections, such as tests of normality and adjustment for multiple comparisons
☐	☒	A full description of the statistical parameters including central tendency (e.g. means) or other basic estimates (e.g. regression coefficient) AND variation (e.g. standard deviation) or associated estimates of uncertainty (e.g. confidence intervals)
☐	☒	For null hypothesis testing, the test statistic (e.g. *F*, *t*, *r*) with confidence intervals, effect sizes, degrees of freedom and *P* value noted<br>*Give P values as exact values whenever suitable.*
☒	☐	For Bayesian analysis, information on the choice of priors and Markov chain Monte Carlo settings
☒	☐	For hierarchical and complex designs, identification of the appropriate level for tests and full reporting of outcomes
☒	☐	Estimates of effect sizes (e.g. Cohen's *d*, Pearson's *r*), indicating how they were calculated

Our web collection on statistics for biologists contains articles on many of the points above.

Software and code

Policy information about availability of computer code

Data collection: Data was collected using the commercial software provided with the instrument: Lumicks Bluelake version 1.7.1

Data analysis: We used Python 3.8 with several libraries for image processing. We used the Laplacian of Gaussian detector from Python's "scipy" for spot detection. We used the Linear Assignment Problem method (Jaqaman et al) and the "scipy" solver "linear_sum_assignment" to do spot tracking. Bleaching trace analysis was done with the "ruptures" library. A full list of the exact python libraries and their versions:
numpy==1.19.5
matplotlib==3.2.2
lumicks-pylake==0.7.1
streamlit==0.74.1
scipy==1.6.1
scikit-image==0.16.2
scikit-learn==0.23.1
pyyaml==5.3.1
pandas==1.0.5
pillow==7.2.0
tifffile==2021.1.11
jupyterlab==2.1.5
notebook==6.0.3
ruptures==1.1.6
pykalman==0.9.5

March 2021

All the code used in this manuscript is available at https://gitlab.tudelft.nl/nynke-dekker-lab/public/cmg-activation. For a more detailed description of the code, see Methods.

For manuscripts utilizing custom algorithms or software that are central to the research but not yet described in published literature, software must be made available to editors and reviewers. We strongly encourage code deposition in a community repository (e.g. GitHub). See the Nature Portfolio guidelines for submitting code & software for further information.

Data

Policy information about availability of data

All manuscripts must include a data availability statement. This statement should provide the following information, where applicable:

- Accession codes, unique identifiers, or web links for publicly available datasets
- A description of any restrictions on data availability
- For clinical datasets or third party data, please ensure that the statement adheres to our policy

Source data are provided with this paper. Raw and processed ensemble and single-molecule data generated in this study have been deposited in the 4TU data repository and can be found at https//doi.org/10.4121/19948253.

Human research participants

Policy information about studies involving human research participants and Sex and Gender in Research.

Reporting on sex and gender	Not applicable
Population characteristics	Not applicable
Recruitment	Not applicable
Ethics oversight	Not applicable

Note that full information on the approval of the study protocol must also be provided in the manuscript.

Field-specific reporting

Please select the one below that is the best fit for your research. If you are not sure, read the appropriate sections before making your selection.

☒ Life sciences ☐ Behavioural & social sciences ☐ Ecological, evolutionary & environmental sciences

For a reference copy of the document with all sections, see nature.com/documents/nr-reporting-summary-flat.pdf

Life sciences study design

All studies must disclose on these points even when the disclosure is negative.

Sample size	Sample size is mentioned in figure for each experiment, and the results of the quantitative analysis of multiple DNA tethers are presented as histograms. We did not conduct any sample size precalculations, but data collection continued until the data set was large enough to perform meaningful statistical analysis of the distribution. This typically required at least 25 individual DNA tethers containing at least 30 fluorescent foci. Sample size is conditioned by the intrinsic low throughput of the optical force/fluorescence instruments, and was selected as a reasonable minimum to perform statistical analysis.
Data exclusions	1) While the distance between the optical traps is constant, the force between the traps can fluctuate; jumps in the force signal could indicate, for instance, DNA 'slipping' from the beads, or a protein aggregate landing on a bead, which makes the location signal inaccurate. Hence, if the force signal exhibits a jump larger than $2\sigma F$ after fitting with CPA, where $\sigma F = 0.1$ pN is the force fluctuation of a clean trace, only the part of the trace before that jump is used for motion analysis. 2) Diffraction-limited spots containing more than 5 fluorescent proteins, likely aggregates, were filtered out. 3) Any traces starting or ending within 1 kbp from a bead are filtered out to prevent any proteins likely stuck to a bead from entering the dataset. 4) Any traces starting after frame 3 are also filtered away because we do not expect any fluorescent protein to land on the DNA during the scan. 5) The last frame of each trace is omitted for motion analysis because photobleaching often happens while that frame is being taken, resulting in a distorted spot with an incorrect position. 6) Finally, in order to perform reliable motion analysis, only traces with a length of 14 frames or more are retained and used for motion analysis.
Replication	When collecting a data set with single-molecule optical force spectroscopy, every DNA-protein complex is trapped and measured independently. For each experimental condition, these independent measurements are repeated as many times as needed to reach the statistics discussed above in "Sample Size", with all other experimental conditions held constant. Even data sets that were collected over the course of one day are the result of dozens of individual experiments performed in an identical fashion on identically-prepared DNA-protein complexes. Each data set was acquired every day over 2-4 weeks, with a new and independent experiment conducted every day. All attempts at replication were successful.
Randomization	Apart from data exclusion process described before, no other experimental groups were further introduced.

Blinding

These experiments were not blinded, but the appropriate positive and negative controls are described in the main text and supplementary information. In general, blinding is not feasible in single-molecule experiments, and targeted controls are preferred. This is because we image a well characterized biochemical system, which we study at higher temporal and spatial resolution with targeted single-molecule experiments. We take the following measures to avoid bias in our single-molecule measurements:
1. In our experiments, we alternate data acquisition between different conditions (ATP or no ATP, etc.); thus, any temporal or instrument-specific fluctuations are included in all the data.
2. The single-molecule data was collected on two separate instruments. One of the main finding of this manuscript (that CMG diffuses in the absence of nucleotide, but that nucleotide binding halts it) was an unexpected one; two independent researchers reached the same conclusions based on data that they collected independently. The novel data analysis that was required to analyze the CMG motion data was furthermore tested on simulated data of experimental traces (Supplementary Figure 6) to identify proper analysis settings.

In experiments other than single-molecule experiments, investigators were not blinded to group allocations during data collection. Nevertheless, we conducted necessary negative and positive controls in every experiment and believe that blinding is not relevant to such experiments.

Reporting for specific materials, systems and methods

We require information from authors about some types of materials, experimental systems and methods used in many studies. Here, indicate whether each material, system or method listed is relevant to your study. If you are not sure if a list item applies to your research, read the appropriate section before selecting a response.

Materials & experimental systems

n/a	Involved in the study
☐	☒ Antibodies
☒	☐ Eukaryotic cell lines
☒	☐ Palaeontology and archaeology
☒	☐ Animals and other organisms
☒	☐ Clinical data
☒	☐ Dual use research of concern

Methods

n/a	Involved in the study
☒	☐ ChIP-seq
☒	☐ Flow cytometry
☒	☐ MRI-based neuroimaging

Antibodies

Antibodies used

M2 anti-flag affinity beads (Merck-Sigma # A2220)

Validation

We used the anti-FLAG beads for the purification of recombinant proteins containing a FLAG tag. These beads were purchased from Merck-Sigma and have been validated by the manufacturer using chemoluminescent and Western blot detection (https://www.sigmaaldrich.com/specification-sheets/120/274/F3165-BULK.pdf)
